# *Candida auris*: a global pathogen that has taken root in Colombia

**DOI:** 10.7705/biomedica.7082

**Published:** 2023-08-31

**Authors:** Patricia Escandón, Shawn R. Lockhart, Nancy A. Chow, Tom M Chiller

**Affiliations:** 1 Grupo de Microbiología, Instituto Nacional de Salud, Bogotá, D.C., Colombia. Instituto Nacional de Salud Bogotá, D.C. Colombia; 2 Mycotic Diseases Branch, Centers for Disease Control and Prevention, U.S. Department of Health and Human Services, Atlanta GA, USA Centers for Disease Control National Center Prevention Service Centers for Disease Control and Prevention Atlanta GA USA

**Keywords:** Candida auris, infections, drug resistance, fungi, whole genome sequencing, Colombia., Candida auris, infecciones, resistencia a medicamentos, hongos, secuenciación completa del genoma, Colombia.

## Abstract

*Candida auris* has been recognized as an emerging multidrug-resistant pathogen with a significant public health burden, causing cases of invasive infection and colonization due to its persistence on inanimate surfaces, ability to colonize skin of some patients, and high transmissibility in healthcare settings.

The first sporadic report of the isolation of this species from the ear canal of a patient in Asia was in 2009 and reports from other regions of the world soon followed. However, it was not until 2015 that global epidemiological alerts were communicated as a result of an increasing number of reports of invasive infections caused by *C. auris* in several countries. Colombia was soon added to this list in 2016 after an unusual increase in the number of *C*. *haemulonii* isolates was reported, later confirmed as *C. auris*. Since the issuing of a national alert by the Colombian National Institute of Health together with the Ministry of Health in 2016, the number of cases reported reached over 2,000 by 2022.

Colombian isolates have not shown pan resistance to available antifungals, unlike *C. auris* strains reported in other regions of the world, which leaves patients in Colombia with therapeutic options for these infections. However, increasing fluconazole resistance is being observed. Whole-genome sequencing of Colombian *C. auris* isolates has enhanced molecular epidemiological data, grouping Colombian isolates in clade IV together with other South American isolates.

Data from Colombia showed that public health authorities, scientific community, and the general public need to be aware of fungal diseases as they present an often-deadly threat to patients.

## Resumen

*Candida auris* ha sido reconocido como un agente patógeno multirresistente emergente con una carga significativa en la salud pública. Genera casos de infección invasiva y colonización debido a su persistencia en superficies inanimadas, su capacidad para colonizar fácilmente la piel de algunos pacientes y su alta transmisibilidad en el ambiente hospitalario.

El primer reporte esporádico de esta especie fue en Asia en el 2009 cuando se realizó su aislamiento a partir del conducto auditivo de un paciente, y pronto le siguieron reportes en otras regiones del mundo. Sin embargo, no fue hasta 2015 que se conocieron las alertas epidemiológicas a nivel mundial debido a un aumento en el número de casos de infecciones causadas por *C. auris* en varios países.

Colombia se sumó a la lista en 2016 luego de un aumento inusual en el número de aislamientos de *C. haemulonii* informados, que luego se confirmaron como *C. auris*. Desde que el Instituto Nacional de Salud junto con el Ministerio de Salud emitieron la Alerta Nacional en el 2016, el número de casos reportados superó los 2.000 en el 2022.

Los aislamientos colombianos no han mostrado resistencia generalizada a los antifúngicos disponibles, contrario a lo reportado para cepas de *C. auris* en algunas regiones del mundo, por lo que los pacientes en Colombia aún cuentan con opciones terapéuticas para estas infecciones. No obstante, se ha observado un aumento en la resistencia al fluconazol. La secuenciación del genoma completo agrupó los aislamientos colombianos en el Ciado IV, junto con otros sudamericanos de *C. auris*, y aportó al conocimiento de los datos epidemiológicos moleculares de esta especie.

Los datos de Colombia evidencian que las autoridades de salud pública, la comunidad científica y el público en general deben ser conscientes de las enfermedades fúngicas, ya que a menudo representan una amenaza mortal para los pacientes.

*Candida auris* is an emerging yeast, first reported over a decade ago and is now a cause of severe invasive infections in healthcare settings worldwide. It is of public health concern because its identification via conventional biochemical methods is difficult. It persists on surfaces and has high transmissibility. Serious bloodstream infections of *C. auris* have been reported globally in healthcare facilities, causing a mortality rate between 30 and 60% ^1^.

In Latin America, bloodstream infections due to *Candida* (candidemia) have an incidence of 1.18 cases per 1000 hospital admissions. In Colombia, the incidence is 1.96 cases per 1000 hospital admissions, one of the highest in the region according to data from the Network of Invasive Mycosis in Latin America ^2^.

Over time, the number of candidemia cases in Colombia has continued to increase, and changes in the distribution of the causative species from C. albicans to *Candida species* other than *C. albicans* have occurred. There has been a predominance of C*. tropicalis*, *C. parapsilosis* and *C. glabrata* cases ^3^ and, more recently, a significant number of cases caused by the multidrug- resistant emerging yeast *C. auris*, with more than 2,000 cases confirmed by the *Instituto Nacional de Salud*^4^^,^^5^.

The clinical importance of *C. auris* has increased in different regions of the world, not only because of the number of patients infected but also because of the antifungal resistance patterns. This is the reason for the heightened clinical concern in some areas. This review presents the general global situation of this fungal pathogen, emphasizing the experience of laboratory- based surveillance in Colombia.

## Current state of *Candida auris*

*Candida auris* was first described in 2009 in Japan where it was isolated from the external ear canal of a patient ^6^. In 2011, three bloodstream infections caused by *C. auris* were described in South Korea ^6^, establishing that this new species could cause invasive infections. Subsequently, there were reports from several hospitals in India ^7^ and South Africa ^8^ confirming the wide distribution of this organism. The first description of *C. auris* in the Americas was in 2012 and 2013 from bloodstream infections in patients from Venezuela, although some publications erroneously described cases as originating from Brazil ^9^. In 2015, studies based on whole-genome sequencing of isolates from these countries suggested nearly simultaneous, and recent, independent emergence of different clonal populations on three continents ^10^.

In the Americas, Colombia was the next country in the region to identify cases dating back to 2013, where bloodstream isolates were initially identified as *Candida haemulonii*, and then confirmed as *C. auris* in 2015 ^4^^,^^5^^,^^11^. These isolates were identified as the South American clade (clade IV).

In 2016, the United States and the Pan American Health Organization (PAHO) issued alerts on *C. auris*. This helped to generate outreach and identification of the further spread in the region. Shortly after these alerts, *C. auris* was reported in the United States, Panama, and Canada. Isolates from the US were found to be from multiple clades, not just the South American clade, while Panama isolates were all South American. It was clear that spread was occurring from travel within the region but also from outside ^10^^,^^12^^,^^13^.

In 2019, two more countries in the Americas reported isolates, Chile and Costa Rica, both with cases identified with travel history ^14^; and then during the COVID-19 pandemic, Brazil and Mexico were added to the list of countries reporting *C. auris* cases ^15^^,^^16^.

**Figure 1.**
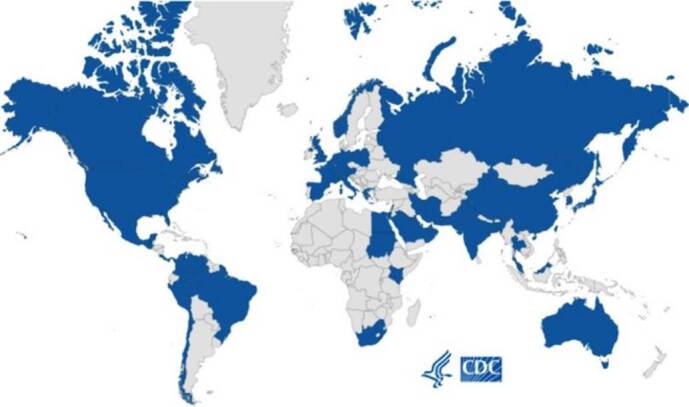
Countries with reported *Candida auris* cases through February 15, 2021

A map published by the Centers for Disease Control and Prevention (CDC) shows the continuous spread of *C. auris* in the United States as well as global transmission (https://www.cdc.gov/fungal/diseases/candidiasis/tracking-c-auris. html, ^17^. Figure 1 shows the global distribution of this emerging pathogen through February 2021, indicating countries with cases of *C. auris*.

*Candida auris* has been recovered from many specimen types, including sterile body fluids (such as urine), tissues, and other sources such as burns and wounds, as well as non-sterile sites such as respiratory specimens and superficial body sites (axilla, groin, nose, etc.). Globally, bloodstream infections are the most common infection associated with *C. auris* invasive disease, leading to elevated mortality rate in patients ^18^^-^^20^.

There are no comprehensive data regarding the specific risk factors for *C. auris* infections compared to colonizations. However, available data suggest that predisposing factors for acquiring an invasive infection by this pathogen are very similar to those of other types of *Candida* infections, including antifungal and antibiotic use, the use of lines and tubes penetrating the body (central venous catheters, feeding tubes), diabetes and surgeries ^20^^,^^21^. The global and local spread of *C. auris* has been enhanced by the ability of the pathogen to survive for long periods of time on environmental surfaces in healthcare settings, especially when infection control and prevention practices are suboptimal. Dispersion, spread, and transmission within and between medical institutions and geographical areas have been facilitated by its capacity to spread through contact with contaminated environmental surfaces or equipment, and potentially from person to person ^4^^,^^10^^,^^21^.

## Laboratory-based surveillance of *Candida auris* in Colombia

In Colombia, the circulation of *C. auris* was retrospectively identified in 2012 in the city of Santa Marta, on the north coast of the country. Although those sporadic isolates were not confirmed as being *C. auris* until 2015, they add to the case count of national surveillance. Laboratory-based surveillance of *C. auris* officially began in 2016, almost simultaneously with the global alerts and the identification of an unusual number of cases of bloodstream infections caused by *C. haemulonii*, later confirmed by molecular methods as *C. auris*, which prompted the laboratory-based surveillance. The identification of *C. auris* in the northern and central regions of Colombia urged public health authorities to issue a national alert for *C. auris* invasive infections ^4^^,^^22^^,^^23^. Subsequently, the epidemiological alert encouraged increased detection and reporting of patients colonized and infected by this pathogen.

Following the issue of the national alert, the *Instituto Nacional de Salud* of Colombia encouraged the national laboratory network to report all infections and outbreaks caused by *C. auris*. This is one of the few fungal diseases with mandatory reporting in the country. Public health laboratories also submit clinical isolates, mainly from invasive specimens (blood, urine with catheter, cerebrospinal fluid, and other sterile fluids) from their local network of hospitals and health institutions to the Microbiology Group at the Instituto Nacional de Salud. All the isolates are confirmed using polymerase chain reaction (PCR) with specific primers ^24^ and Biotyper MALDI-TOF ((BrukerTM, Billerica, MA, USA) at the reference laboratory of the Instituto Nacional de Salud.

**Figure 2.**
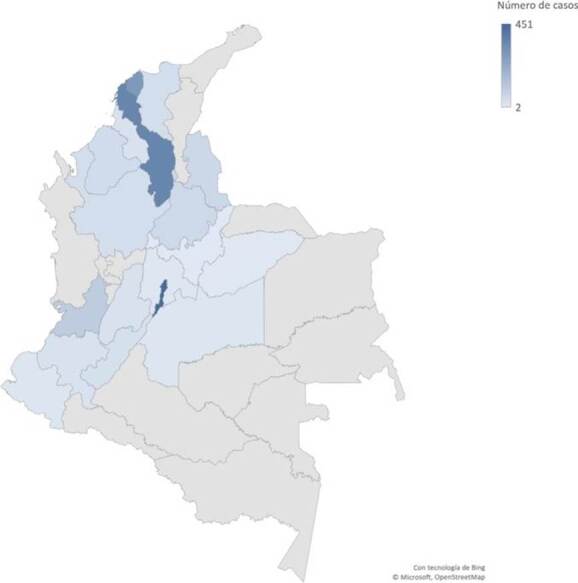
Confirmed cases of *Candida auris* in Colombia, 2016-2022. Darked blue color regions represent those areas with higher numbers of cases.

From 2016 to 2022, a total of 2,119 isolates were received through laboratory-based surveillance, including 393 from colonization (18.5%) and 1,726 clinical cases (81.5%). The distribution of reported cases in Colombia varies within the 18 out of 33 departments participating in the surveillance. Bogotá (21.3%), Bolívar (16.7%) and Atlántico (13.7%) submitted the highest number of isolates (figure 2). The median age of patients was 34 years, but children (less than 15 years old) comprised 22% of the total cases, and pediatric intensive care units have played an important role in the transmission of *C. auris* in hospital settings. Most cases (67.3%) occurred in men. As has been reported globally, clinical isolates recovered from Colombian patients were mainly from bloodstream infections (78.6%) and a smaller proportion from other sterile body fluids, urine specimens, and other non-sterile specimens ^4^^,^^5^^,^^25^^-^^27^. There have been many reports of *C. auris* infections associated with COVID-19 patients ^15^^-^^17^^,^^28^, and this was also true in Colombia, where 189 (25.2%) patients with a confirmed diagnosis of SARS-CoV-2 also had an infection with *C. auris* during the period 20202021.

*Candida auris* is often misidentified when using the standard diagnostic tests available in Colombian laboratories, which can lead to inaccurate treatment of patients. In Colombia, the availability of advanced diagnostic technologies such as MALDI-TOF is not common in most public laboratories, so routine diagnosis is performed using automated equipment such as VITEK® 2 (Biomerieux). Laboratory-based surveillance in Colombia often receives isolates wrongly identified as *C. auris* by VITEK® 2. The most frequent misidentification of *C. auris* is as *C. haemulonii*, as expected from its close phylogenetic relationship (22% of the isolates from 2018-2021 were identified as *C. haemulonii*).

Given this finding, the Microbiology Group of the *Instituto Nacional de Salud* carried out a retrospective analysis of the isolates reported through the World Health Organization net as *C. haemulonii* and found that during the period 2013-2018, a total of 448 isolates of *C. haemulonii* had been reported, mainly from Barranquilla (52%), Santander (14.5%), Bogotá (13%) and Valle del Cauca (7%). This finding suggests that previously undetected *C. auris* may have been circulating in Colombia before 2016 and may have been misidentified as its closely related species C. haemulonii ^25^^-^^27^, which is not as common ^5^.

Another troubling feature of *C. auris* is that it can colonize patients for many months and persist in the environment despite the use of standard healthcare facility disinfectants. Patients may be asymptomatically colonized with *C. auris* on the skin, including nares, oropharynx, rectum, axilla, and other body sites, and this risks transmission of the fungus to other patients within the same healthcare setting, putting all the patients at risk for invasive *C. auris* infections. The Centers for Disease Control and Prevention (CDC) recommends screening for *C. auris* as an important component of surveillance and outbreak investigations.

In Colombia, several outbreaks have been identified by the national surveillance system since the first cases were reported through the laboratorybased surveillance in 2016. The first outbreaks in different facilities characterized by the *Instituto Nacional de Salud* together with the support and effort of personnel from the Mycotic Disease Branch at CDC occurred in four hospitals in three cities with 40 confirmed cases of *C. auris* candidemia.

Investigation of these cases found associated risk factors similar to those reported for infections by other *Candida species* (diabetes, hemodialysis, catheter use), high mortality rates (up to 50%), and hospital transmission, confirming the ability of this fungus to cause healthcare-associated outbreaks ^21^^,^^28^.

A recent study on the north coast of Colombia, designed to compare mortality rates of *C. auris* candidemia with those of the other *Candida species*, revealed that the mortality of *C. auris* candidemia was not higher than that of other *Candida species*, reinforcing the need for adherence to prevention and control practices in the hospital setting ^29^^,^^30^.

## Molecular epidemiology and whole-genome sequencing of *Candida auris*

The molecular epidemiology of *C. auris* is complex. From its first genomic description, four major subtypes or clades (clades l-IV) were identified, displaying a strong association with geography ^10^. Clade I was characterized by *C. auris* cases from countries in South Asia (i.e., India and Pakistan), clade II by a case from Japan in East Asia, clade III by cases from South Africa in Africa, and clade IV by cases from Venezuela in South America. With tens to hundreds of thousands of single-nucleotide polymorphisms (SNP) separating the clades, clades l-IV were reported to be genetically distinct, with significantly less diversity observed within each clade. More recently, a description of Iranian cases resulted in a fifth major clade of *C. auris* (clade V) ^31^^,^^32^.

**Figure 3.**
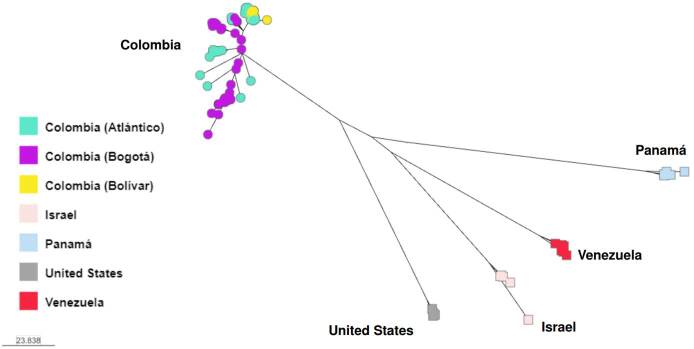
Whole genome sequencing of Colombian *Candida auris* isolates recovered from 2016 until 2021. Isolates cluster by country, except 16 isolates from Bogotá that grouped with isolates from Venezuela. Analysis was done using MycoSNP geneflow, version 0.21, and B11245 (Venezuela) Clade IV reference genome.

In response to the 2015-2016 *C. auris* outbreaks in Colombia, whole genome sequencing was conducted to describe its molecular epidemiology ^11^. Cases were from clade IV and clustered with Venezuela cases, further supporting the strong phylogeographic structure of *C. auris*. Additionally, *C. auris* was isolated from the healthcare environment (e.g., bed rails, mobile equipment, floors, door handles). Isolates also belonged to the clade IV. Although cases and environmental samples represented four hospitals from two distinct geographic areas, isolates were separated by only 40 SNPs. Notably, a well-supported separation was observed between cases and environmental samples from the northern region compared to those from the central region. These findings provided evidence for localized transmission of *C. auris* at the regional level as well as a widespread outbreak. As part of an updated molecular epidemiologic description, we analyzed *C. auris* cases across Colombia, occurring from 2016 until 2021, and isolates continued to be of clade IV (unpublished data, figure 3). This finding suggests that *C. auris* introduction of other clades into Colombia has not occurred or has occurred but without subsequent transmission.

Globally, over 40 countries now report *C. auris* cases, and several countries have reported cases from multiple clades including Canada, Kenya, Mexico, South Africa, and the United States ^33^^-^^39^. Since the first genomic descriptions ^10^^,^^34^, genetic diversity is increasing within the clades. It is of interest to understand how the global distribution of C. auris will affect this species in Colombia. Future investigations should frequently monitor the molecular epidemiology of *C. auris* across the country to better understand changes in spread, antifungal resistance, and epidemiology.

## *Candida auris* and antifungal resistance

Antifungal resistance has been one of the defining characteristics of *C. auris*, but the story in Colombia has been slightly different. Since the first reported cases of Colombia in 2015 and using the preliminary breakpoints set by the CDC, the resistance rate to fluconazole was 11% (10/87 isolates) and to amphotericin B was 31% (27/87 isolates) ^4^^,^^11^. This lack of resistance is consistent with US findings in Chicago among isolates whose closest match were Colombian clade IV isolates (Centers for Disease Control and Prevention, unpublished data; 33, 34). It contrasts with some of the first clade IV isolates from Venezuela and Panama, which are universally resistant to fluconazole, and isolates from other clades with higher azole resistance ^9^^,^^12^.

Other Colombian studies with early emergent isolates showed the same sensitivity pattern, with fluconazole sensibility ranging from 18 to 59% and amphotericin B resistance ranging from 22 to 29% ^18^^,^^22^. Amphotericin B-resistant isolates were more common in the north of the country ^3^^,^^12^. Throughout the surveillance period, echinocandin resistance was extremely rare, with only one isolate identified ^9^^,^^10^. Some evidence points out increased fluconazole resistance in Colombia. From 2016 through 2019, the fluconazole minimum inhibitory concentration 50 (MIC_50_) was 4-8 µg/ml, but in 2020, it increased to 32 µg/ml, indicating that at least 50% of the isolates from 2020 were fluconazoleresistant ^9^. During the same time frame, a similar change in the MIC_50_ to amphotericin B was not observed, but the overall resistance rate from 2016-2020 increased to 33%.

Pediatric cases of *C. auris* have been rare in Colombia, with only two reports of 13 and 6 cases from two facilities ^11^^,^^12^. Among those 19 cases, 3 (16%) and 13 (68%) were resistant to fluconazole and amphotericin B, respectively. The only echinocandin-resistant isolate was from a pediatric patient ^20^.

The fluconazole-resistance mechanism of Colombian isolates is predominantly the amino acid change in the ERG11 gene at either Y132F or K143R, similar to that of clade 1 isolates ^11^^,^^40^^,^^41^. The amphotericin B-resistance mechanism is still unknown. In a single Colombian study monitoring the outcome of 34 *C. auris* patients, antifungal resistance was not statistically associated with patient outcome ^21^.

## Final remarks

As *C. auris* emerged on the global scene, it almost immediately caused a significant number of outbreaks and captured the attention of both the scientific community and public health authorities. The impact was such that it was recently added to the WHO fungal priority pathogens list in the critical priority group. This classification is meant to highlight the critical need to do further research and develop diagnostics tools and treatments ^42^.

What lies in the future for emerging fungal pathogens is uncertain. We still do not understand how *C. auris* emerged so rapidly as a multidrug-resistant, adaptive, and transmissible fungus with an unprecedented capacity to survive in hospital environments and cause infections in severely ill patients.

*Candida auris* is here to stay, with a high number of cases in Colombia, the region, and the world. This organism highlights the need for fungal surveillance to detect the emergence of new fungal pathogens. On the positive side, *C. auris* has brought an unprecedented awareness of fungal diseases and the need for enhanced detection and diagnosis.
